# The Associations between Cognitive Function, Depressive Symptoms, and Contact with Adult Children in Older Couples

**DOI:** 10.3390/jcm12165431

**Published:** 2023-08-21

**Authors:** Jin-young Min, Beom Kim, Kyoung-bok Min

**Affiliations:** 1 Veterans Medical Research Institute, Veterans Health Service Medical Center, Seoul 05368, Republic of Korea; minjy@bohun.or.kr; 2 Department of Internal Medicine, Veterans Health Service Medical Center, Seoul 05368, Republic of Korea; glom@hanafos.com; 3 Institute of Health Policy and Management, Seoul National University Medical Research Center, Seoul 03080, Republic of Korea; 4 Department of Preventive Medicine, College of Medicine, Seoul National University, Seoul 03080, Republic of Korea

**Keywords:** older couple, cognition, depression, family relationship, sex difference

## Abstract

The aim of this study was to investigate the association between cognitive function and depressive symptoms in older couples while also examining the relationship between cognitive performance and the frequency of contact with adult children. A total of 96 couples volunteered for this study and provided their informed consent at enrollment. Participants completed a neuropsychological test battery consisting of five cognitive domains: attention, language and related functions, visuospatial functions, memory, and frontal/executive functions. Symptoms of depression were assessed using the short version of the Geriatric Depression Scale. The number of contacts with children was categorized into ≥1 per month and <1 per month. We found that the cognitive functions of husbands with depressed wives were significantly lower in the frontal/executive functions. In contrast, the wives’ cognitive performance was not associated with the husbands’ depressive symptoms. For couples who had contact with their adult children less than once a month, the odds of the husbands with lower cognitive performance were significantly higher, which was reflected in their scores in visuospatial and executive functions. Among older married couples, the cognitive functions of husbands may be influenced more by their wives’ mental health and degree of contact with their adult children. This infers that wives and offspring may act as a buffer against the cognitive impairment of older married men.

## 1. Introduction

As people age, cognitive function is an important indicator of health and mortality risk and the ability to maintain an independent physical life [1]. Several studies have documented factors that are significantly linked to the cognitive function of older adults [2,3]. Some of these factors (both risk and protective) include advanced age, female sex, educational attainment, marital status, exercise, social engagement, mental or physical diseases, and the apolipoprotein E gene [2,3].

An important factor affecting cognitive function is family members [4]. Family members are a group of people bound by blood, marriage, or adoption and include spouses, parents, children, siblings, and siblings-in-law. Family members often share structural, associational, functional, affectual, consensual, and normative relationships, which help shape individuals’ cognition [4,5,6]. Family members may have more importance for older people because, as individuals age, their other social connections become less central in their lives, and their need for caregiving increases [7]. Among older adults, those who had a large number of family members [8], frequent contact with their children [9], intergenerational support from children [10], or downward family support (e.g., grandparental child care) performed better on cognitive, executive function [11], and verbal fluency tests [12]. Among family members, the spouse may have the greatest effect on their partners’ cognitive function. Spouses’ emotional, intellectual, and daily life activities are closely intertwined; thus, each partner’s attributes affect the other partners’ health outcomes, including cognitive health [13]. Many studies have suggested that spousal strain, chronic illnesses, physical disability, and bereavement were associated with their partners’ cognitive impairment and decline [14,15,16]. 

The association between depression and cognition across partners may be particularly strong among older couples because emotional investments in the marriage relationship and partner often increase over time. Whether there are sex differences in the degree of association between depression and cognition across partners is a question that has not been sufficiently or consistently answered. Some studies have reported that more severe depression in husbands is significantly associated with lower cognitive scores for their wives, but not vice versa [17,18]. Gerstorf et al. (2009) found that wives’ depressive symptoms can predict their husbands’ memory decline [19]. However, Lee et al. (2012) suggested that the emotional health of one spouse influenced the health of the other but does not influence the other’s cognitive health [20]. 

Increasing age is associated with a steeper decline in cognitive function [1]. Poor cognitive performance is associated with adverse health outcomes, including physical and mental diseases and mortality [1,2,3]. Thus, understanding the positive contributions of cognitive decline and maximizing the benefits during aging may be an essential topic for individuals and public health fields. Considering that older adults’ cognitive function is affected by partners’ health status [14,15,16] or family and intimate relationships [9,10,21], and the effect may be different between husbands and wives [13], it is crucial to address family-related factors with positive aspects of older adults’ cognitive health. However, the results on the association between spouses’ depression and partners’ cognitive performance are inconsistent [17,18,19,20]. Evidence on the association between parents’ cognitive function and their children’s contact is limited. 

In the current study, our research question is whether, among older couples, spouses’ depressive symptoms are associated with one individual’s cognitive function and the relationship with their adult children. We recruited older married couples and analyzed the cross-partner association between depressive symptoms and cognitive function (which we assessed by testing attention, language and related functions, visuospatial functions, memory, and frontal/executive functions). We further examined the association between the cognitive performance of couples and the frequency of contact with their adult children.

## 2. Materials and Methods

### 2.1. Study Population

**Figure 1:**
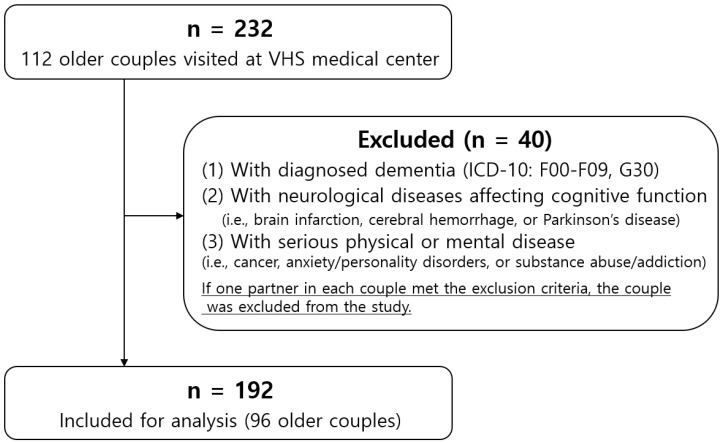
Flow chart of the study population.

This study is a hospital-based survey conducted at the Veterans Medical Research Institute in the Veterans Health Service Medical Center in Seoul, Korea. The Veterans Medical Research Institute has researchers, places, and equipment to conduct interviews and medical examinations for clinical studies. A total of 232 participants (116 couples) volunteered between 29 March and 30 September 2021. Among these patients, those included in the study were patients who: (1) visited the Department of Neurology with complaints of cognitive decline; (2) had no difficulty or inability in performing daily activities, including doing heavy and light housework, shopping, preparing meals, and managing money; (3) were able to complete a neuropsychological evaluation and questionnaires independently; and (4) had agreed to participate in this study. If both partners in each couple met the inclusion criteria, they were included as the target population. Those excluded from the study were patients who: (1) had been diagnosed with dementia (ICD-10: F00-F09, G30) and other neurological diseases affecting cognitive function (i.e., brain infarction, cerebral hemorrhage, or Parkinson’s disease); and (2) were currently suffering from a serious physical or mental disease or diseases (e.g., cancer, anxiety/personality disorders, or substance abuse/addiction). The inclusion and exclusion criteria for clinical conditions were evaluated by experienced neurological clinicians. If one partner in each couple met the exclusion criteria, the couple was excluded from the study. Forty participants were finally excluded if they had dementia, neurological disorders, serious physical/mental diseases, or their spouse was suffering (Figure 1). Our study population was 192 participants (96 couples). 

### 2.2. Ethical Consideration

The study protocols were approved by the Institutional Ethical Review Board of the Veterans Health Service Medical Center (IRB no. BOHUN 2021-02-024, BOHUN 2021-01-066). All participants provided signed informed consent prior to study enrollment. To guarantee the autonomy of the subjects participating in the study, the purpose of the study, the data collection process, anonymity, and confidentiality were explained to all participants. The data and private information collected for the study were kept anonymous, the Personal Information Protection Act kept confidential, and all information that could identify the participants was deleted after the study was completed.

### 2.3. Neuropsychological Evaluation

Participants completed the brief version of the Seoul Neuropsychological Screening Battery (SNSB), which is called the SNSB-Core (SNSB-C) [22]. The SNSB-C is a comprehensive test that evaluates the level of cognitive function or impairment in five cognitive domains: attention, language and related functions, visuospatial functions, memory, and frontal/executive functions [22]. The SNSB-C includes the Korean-Boston Naming Test (K-BNT), Rey Complex Figure Test (RCFT), Rey Complex Figure Test (SVLT), Digit Span Substitution Test (DSST), Controlled Oral Word Association Test (COWA) with animal naming and semantic fluency test (‘ㄱ’), Korean Trail Making Test (KTMT), and the Color Word Stroop Test (COWAT). The composite scores of the SNSB-C were expressed as a percentile standardized for age, sex, and education. A higher percentile score indicates better cognition. Participants scoring ≤ 16th percentile were considered cognitively impaired [23,24].

### 2.4. Depression Assessment

Symptoms of depression were assessed using the short version of the Geriatric Depression Scale (GDS). The GDS is a self-reported measure of depression in older adults, with the short version consisting of 15 questions derived from the original 30-question version. It was designed to assess depressive symptomatology in older people and excludes any questions relating to the physical symptoms of depression common in old age. A trained psychologist read out each question, which was designed to elicit a “yes” or “no” response and to measure how the patient felt over the past week. All questions were asked with no further explanation or elaboration. Each answer that indicated depression was scored as one point. Scores greater than five indicated probable depression [25,26].

### 2.5. Contact with Children

Each participant was asked, “Do you have any living children?” with responses of “yes” or “no”. If the response was “yes”, we further asked, “How many times have you contacted your child in the past month?” A response of “more than one contact with children within a month” was scored as 1, while less than or equal to 1 contact per month was scored as 0.

### 2.6. Confounding Variables

We collected data for the following demographic variables: age, years of education, and monthly income (<1,000,000 Korean won, 1,000,000~1,999,000 Korean won, 2,000,000~2,999,000 Korean won, 3,000,000~4,999,000 Korean won, and ≥5,000,000 Korean won). We also surveyed health-related behavior, including current smoking status, alcohol drinking status, and engagement in moderate physical activity. Finally, the medical condition of each participant was assessed in terms of the presence or absence of hypertension, dyslipidemia, and type 2 diabetes. 

### 2.7. Statistical Analysis

Continuous and categorical data of husbands and wives were compared using the *t*-test and the Chi-square test, respectively. Multiple logistic regression was performed to estimate the odds ratio (OR) and its 95% confidence interval (CI) for cognitive impairment, where cognitive impairment was defined as a ≤16th percentile score on each SNSB-C test item. We also conducted the multiple linear regression analysis to estimate the association of cognitive performance with spousal depressive scores or frequency of contact with adult children. The dependent variable was the husbands’ or wives’ depression and frequency of contact with their adult children. The independent variables were the husbands’ or wives’ cognitive performance and confounding variables. These regression models were adjusted for age, income, education, cigarette smoking, alcohol drinking, engagement in moderate physical activity, and the patient’s history of hypertension, dyslipidemia, and type 2 diabetes. All analyses were performed using the Statistical Analysis System software version 9.2 (SAS Institute, Cary, NC, USA), and statistical significance was set at *p* ≤ 0.05.

## 3. Results

### 3.1. Participants’ Characteristics

Table 1 shows the demographic characteristics, health behavior, medical conditions, and variables of interest of the study population, which consisted of 96 older couples (n = 192). Husbands were significantly older than wives (75.47 years vs. 72.10 years, *p* < 0.001). The husbands’ mean number of educational years was 11.63 years, which was significantly higher than that of wives. There was no difference in the mean monthly incomes of husbands and wives. Husbands had higher proportions of current smokers or drinkers, whereas husbands and wives did not differ in their engagement in moderate physical activities. In terms of disease histories, a higher proportion of wives had hyperlipidemia (57.41% vs. 42.59%). In contrast, husbands and wives did not differ in their geriatric depression scores and their respective proportions displaying depressive symptoms. Approximately half of all subjects (both husbands and wives) reported contacting their children more than once a month.

### 3.2. Comparison of Neuropsychological Test Scores between Husbands and Wives

Table 2 compares husbands’ and wives’ neuropsychological test scores. Except for visuospatial function (RCFT test), husbands and wives differed significantly in their neuropsychological test scores. Husbands’ test scores (60.96) in the K-BNT (language test) were significantly higher than those of the wives (47.99). Conversely, the wives’ test scores in the total SNSB-C and SVLT (memory test) were significantly higher than those of the husbands: 52.67 vs. 39.98 for total scores of SNSB-C and 58.64 vs. 39.48 for SVLT. In the front-executive tests, the wives’ scores in the DSST (60.62 vs. 49.76) and CWST (50.60 vs. 38.28) were significantly higher than those of the husbands. Husbands’ and wives’ COWAT and KTMT test scores did not differ significantly. 

### 3.3. Association between Older Couples’ Cognitive Impairment and Spouses’ Depressive Symptoms

Table 3 lists the OR values for cognitive impairment in relation to the depressive symptoms of spouses. The results show that the cognitive functions of husbands with depressed wives were significantly lower in the total scores of SNSB-C (OR = 5.65; 95% CI: 1.42–22.49), COWAT: animal (OR = 3.96; 95% CI: 1.15–13.67), COWAT: animal+ “ㄱ” (OR = 6.27; 95% CI: 1.54–25.59), and KTMT (OR = 26.64; 95% CI: 1.09–654.01). In contrast, the wives’ cognitive performance was not associated with the husbands’ depressive symptoms.

Table 4 shows the beta coefficients for cognitive impairment in relation to the depressive symptoms of spouses. The results show that the cognitive scores of husbands with depressed wives were significantly reduced in the total scores of SNSB-C (beta = −2.472; *p*-value = 0.0063), K-BNT (beta = −2.428; *p*-value = 0.0072), COWAT: animal (beta = −2.235; *p*-value = 0.0138), COWAT: “ㄱ” (beta = −2.110; *p*-value = 0.0249), and COWAT: animal+ “ㄱ” (beta = −2.384; *p*-value = 0.0091). In contrast, the wives’ cognitive performance was not associated with the husbands’ depressive symptoms.

### 3.4. Association between Older Couples’ Cognitive Impairment and Contact Frequency with Their Adult Children

Table 5 shows the OR values for cognitive impairment of older couples in relation to their contact frequency with their adult children. For couples who had contact with their adult children more than once a month, the cognitive functions of both husbands and wives showed no apparent relationship to contact with adult children. However, for couples who had contact with their adult children for less than once a month, the odds of the husbands with cognitive impairment were significantly higher, which was reflected in their scores in the total scores of SNSB-C (OR = 3.82; 95% CI: 1.06–13.75), RCFT (OR = 6.43; 95% CI: 1.01–41.10), COWAT: animal (OR = 6.04; 95% CI: 1.08–33.69), and COWAT: animal+ “ㄱ” (OR = 4.87; 95% CI: 1.04–22.78). In contrast, having less than one contact a month with their adult children had no association with wives’ cognitive performance.

Table 6 shows the beta coefficients for the cognitive performance of older couples in relation to their contact frequency with their adult children. For couples who had contact with their adult children more than once a month, the cognitive functions of both husbands and wives showed no apparent relationship to contact with adult children. However, for couples who had contact with their adult children for less than once a month, the cognitive scores of the husbands were significantly reduced, which was reflected in their scores in the total scores of SNSB-C (beta = −2.649; *p*-value = 0.0074), K-BNT (beta = −2.371; *p*-value = 0.0206), COWAT: animal (beta = −2.083; *p*-value = 0.0354), COWAT: “ㄱ” (beta = −2.302; *p*-value = 0.0242), and COWAT: animal+ “ㄱ” (beta = −2.418; *p*-value = 0.0165). In contrast, having less than one contact a month with their adult children had no association with wives’ cognitive performance.

## 4. Discussion

The present study describes the association between spouses’ depressive symptoms and their partners’ cognitive function among older married couples. Within couples, we also examined whether husbands’ or wives’ cognitive functions were associated with the frequency of contact with their adult children. This infers that wives and offspring may be important factors against the cognitive impairment of older married men.

The present findings suggest a significant association between the specific cognitive tasks of one spouse to the depressive symptoms of the partners, and this association differs between wives and husbands. In a study of 1599 married couples in the Asset and Health Dynamics Among the Oldest Old, Gerstorf et al. (2009) found that wives’ depressive symptoms were associated with greater memory decline in their husbands, while the husbands’ depressive symptoms were associated with better memory function in their wives [19]. In a longitudinal study of 279 older Hispanic couples, Hinton et al. (2009) found that more severe depression among husbands is associated with lower cognitive function in both husbands and wives; however, wives’ cognitive function is influenced by both their own and their partners’ level of baseline depression [17]. In a longitudinal study of aging among 2684 older Korean couples, Lee et al. (2012) found that, on average, a spouse’s cognitive functioning and depressive symptoms significantly affected those of the partner, but depressive symptoms did not predict a partner’s cognitive functioning or vice versa [20]. In a Cardiovascular Health Study of 1028 community-dwelling, African American, older married couples, Monin et al. (2018) found that one spouse’s more severe depressive symptoms tend to predict the other’s lower cognitive function; however, one spouse’s lower cognitive function does not necessarily predict the other’s greater depressive symptoms over time [21]. The inconsistent results may be attributed to differences in the study samples (e.g., different age groups) as well as variations in measurement techniques and methodologies (e.g., the use of different psychiatric measuring approaches, the self-reporting of cognitive symptoms, and the use of objective cognitive measures). The cultural context, especially with regard to asymmetric gender roles, also likely contributes to this inconsistency. Despite the small effect of depression on spouses, the relationship shared by older couples has lasted over an extended period, and therefore, the effect is likely cumulative [21]. 

Why was husbands’ cognitive function more associated with their wives’ depressive symptoms? The results of our study are unable to explain this lack of symmetry between husbands and wives fully. However, two possible explanations are the differences in gender roles and the changing relationships experienced by families later in life. Older adulthood is a period of multiple transitions (e.g., retirement, death of loved ones, and the emptying of the familial nest) [27]. Through the course of life, older adults are more likely to experience decreased social contact with people who previously shared their social domains, resulting in increased social isolation and loneliness [28,29]. The impact of such loneliness may be greater on husbands. For example, retirement is a more drastic social transition among men compared to women. Noh et al. (2019) reported that in the Korean population, the effect of retirement differed by sex, i.e., retirement tended to negatively affect men’s health compared to that of women [30]. Furthermore, women tend to have a larger and more multifaceted network of friends, whereas men tend to focus on close, intimate relationships with only a few people, mainly their spouses [31]. The results of our study are consistent with this view and may indicate that husbands are more emotionally dependent on their wives as they grow older; thus, husbands tend to be more strongly associated with their spouse’s depression. 

Interestingly, sex-based asymmetry in the cognitive effect of one spouse’s depression within older couples was also observed in the effect of their relationships with adult children. Most adult children are closely involved with their aging parents and are pivotal members of their parents’ social networks [32,33]. Having adult children by itself has been shown to benefit parents’ cognitive skills [34]. A higher frequency of contact with adult children improves parental cognitive performance and has been longitudinally linked to slower cognitive decline [35,36]. According to the results of the Korean Longitudinal Study of Aging, frequent contact with children by phone or letters was associated with a reduced risk of cognitive decline over a four-year period [37]. Li et al. (2018) found differential impacts of social networks on the cognitive functioning of older male and female adults; specifically, men tend to benefit more from a higher volume of contact and emotional closeness [38]. Consistent with our results, only the husbands within an older couple show a significant association between a higher frequency of contact with adult children and better cognitive performance. Within a traditional marriage, men’s health tends to benefit more than that of women, and men are more likely to receive emotional support from their spouses than women [39]. In contrast, women tend to carry a disproportionately higher share of the burden of child care, and they are also often under greater parental pressure [40]. Traditional sex roles and family structure impose different pressures and meanings on men and women (i.e., husbands and wives), and this is also reflected in the effects of their relationships with their children. This study has several limitations. The most important limitation is that since our study was conducted with a small size of our study population who visited in Department of Neurology, the results may need to be more generalizable to the older population. Second, the connection between depressive symptoms and cognitive function within couples also depends on the quality of the marriage relationship and the spouses’ satisfaction with the relationship [41,42]. Our study did not consider these variables. Third, sex differences may be explained by parental roles, family maladjustment, and cultural benefits, which we did not consider in this study. Fourth, this is based on a cross-sectional study, and therefore, the robustness of our conclusion needs to be tested more comprehensively. These limitations should be addressed by performing a longitudinal study using a large sample population and accounting for important confounding variables.

## 5. Conclusions

We found that among older couples, husbands’ cognitive performance was significantly associated with wives’ depression and higher contact with adult children, but such an association was not observed for wives. Our data suggest that the positive mental health of wives and frequent contact with adult children may be important factors for the cognitive function of husbands. However, the results would not necessarily be conclusive due to the very small and likely not representative sample and cross-sectional design. Thus, future longitudinal research studies with a large sample are needed to confirm the observed association.

## Figures and Tables

**Table 1:** Comparison of husbands’ and wives’ characteristics among 96 older couples.

Characteristics	Husbands		Wives		*p*-Value
Demographic variables					
Age (year), mean (SD)	75.47	(5.74)	72.10	(5.51)	<0.001
Education year, mean (SD)	11.63	(4.64)	9.57	(4.48)	0.0021
Monthly income (Korean won), no (%)					
<1,000,000	18	(43.90)	23	(56.10)	0.8752
1,000,000~1,999,000	24	(48.98)	25	(51.02)	
2,000,000~2,999,000	19	(50.00)	19	(50.00)	
3,000,000~4,999,000	20	(55.56)	16	(44.44)	
≥5,000,000	15	(53.57)	13	(46.43)	
Health behavior					
Current smoking, no (%)					0.0235
Yes	5	(100.00)	0	(0.00)	
No	91	(48.66)	96	(51.34)	
Alcohol drinking, no (%)					<0.001
Yes	76	(69.72)	33	(30.28)	
No	20	(24.10)	63	(75.90)	
Moderate physical activity, no (%)					0.8847
Yes	43	(49.43)	44	(50.57)	
No	53	(50.48)	52	(49.52)	
Medical condition					
History of disease, no (%)					
Hypertension	55	(51.89)	51	(48.11)	0.5616
Hyperlipidemia	46	(42.59)	62	(57.41)	0.0199
Diabetes mellitus	76	(48.72)	80	(51.28)	0.4595
Variables of interest					
Geriatric depression score, mean (SD)	5.43	(0.43)	5.67	(0.38)	0.6774
Depressive symptoms, no (%)					
Yes	28	(50.91)	27	(49.09)	0.8732
No	68	(49.64)	69	(50.36)	
Number of contact with adult children, no (%)					0.6093
More than once a month	72	(48.98)	75	(51.02)	
less than once a month	24	(53.33)	21	(46.67)	

**Table 2:** Comparison of husbands’ and wives’ neuropsychological test scores mean (SD).

Test Items	Husbands		Wives		*p*-Value
	Mean	(SD)	Mean	(SD)	
Total scores of SNSB-C	39.98	(3.03)	52.67	(3.06)	0.0036
Language					
K-BNT	60.96	(3.16)	47.99	(2.68)	0.002
Visuospatial function					
RCFT	49.20	(3.04)	51.18	(3.09)	0.6485
Memory					
SVLT	39.48	(3.20)	58.64	(2.97)	<0.0001
Frontal-executive function					
DSST	49.76	(3.18)	60.62	(2.98)	0.0135
COWAT: animal	42.33	(2.97)	44.74	(2.74)	0.5522
COWAT: phonemic (‘ㄱ’)	53.46	(3.22)	56.00	(3.05)	0.5668
COWAT: animal+ phonemic (‘ㄱ’)	47.60	(3.04)	50.66	(2.91)	0.4683
KTMT	58.64	(2.46)	56.07	(2.60)	0.4742
CWST	38.28	(2.82)	50.60	(3.18)	0.0042

K-BNT: Korean version of the Boston Naming Test, RCFT: Rey Complex Figure Test, SVLT: Seoul Verbal Learning Test, DSST: Digit Symbol Substitution Test, COWAT: Controlled Oral Word Association Test, K-TMT: Korean version of the Trail Making Test, CWST: Color Word Stroop Test.

**Table 3:** OR for cognitive impairment by spouses’ depressive symptoms among older couples.

Neuropsychological Tests	Cognitive Impairment of Husbands with Depressed Wives			Cognitive Impairment of Wives with Depressed Husbands		
	No. of Subjects with Lower Performance	OR	(95% (CI))	No. of Subjects with Lower Performance	OR	(95% (CI))
Total scores of SNSB-C	28	5.65	(1.42–22.49)	14	1.37	(0.36–5.16)
K-BNT	14	2.17	(0.46–10.23)	13	1.52	(0.26–8.95)
RCFT	18	3.03	(0.60–15.28)	21	1.24	(0.42–3.73)
SVLT	28	0.90	(0.26–3.17)	12	0.61	(0.13–2.88)
DSST	19	0.73	(0.19–2.84)	7	1.13	(0.10–12.37)
COWAT: animal	25	3.96	(1.15–13.67)	17	1.16	(0.33–4.14)
COWAT: ‘ㄱ’	20	3.96	(0.94–16.66)	11	1.40	(0.30–6.49)
COWAT: animal+ ‘ㄱ’	19	6.27	(1.54–25.59)	16	1.92	(0.55–6.66)
KTMT	6	26.64	(1.09–654.01)	11	1.77	(0.36–8.81)
CWST	20	1.26	(0.31–5.08)	16	1.24	(0.34–4.59)

K-BNT: Korean version of the Boston Naming Test, RCFT: Rey Complex Figure Test, SVLT: Seoul Verbal Learning Test, DSST: Digit Symbol Substitution Test, COWAT: Controlled Oral Word Association Test, K-TMT: Korean version of the Trail Making Test, CWST: Color Word Stroop Test.

**Table 4:** Beta coefficients for cognitive performance by spouses’ depressive symptoms among older couples.

Neuropsychological Tests	Cognitive Impairment of Husbands with Depressed Wives			Cognitive Impairment of Wives with Depressed Husbands		
	Beta	SE	*p*-Value	Beta	SE	*p*-Value
Total scores of SNSB-C	−2.473	0.883	0.0063	0.545	0.740	0.4635
K-BNT	−2.428	0.882	0.0072	−0.258	0.642	0.6891
RCFT	0.255	0.912	0.7804	0.483	0.779	0.5366
SVLT	−1.539	0.942	0.1061	0.722	0.708	0.3109
DSST	−0.680	0.952	0.4774	0.577	0.707	0.4168
COWAT: animal	−2.235	0.888	0.0138	0.378	0.667	0.5722
COWAT: ‘ㄱ’	−2.110	0.923	0.0249	−0.835	0.763	0.2769
COWAT: animal+ ‘ㄱ’	−2.384	0.893	0.0091	−0.258	0.725	0.7224
KTMT	−1.073	0.878	0.2251	0.426	0.709	0.5498
CWST	0.104	0.846	0.9025	−0.542	0.799	0.499

K-BNT: Korean version of the Boston Naming Test, RCFT: Rey Complex Figure Test, SVLT: Seoul Verbal Learning Test, DSST: Digit Symbol Substitution Test, COWAT: Controlled Oral Word Association Test, K-TMT: Korean version of the Trail Making Test, CWST: Color Word Stroop Test.

**Table 5:** OR for cognitive impairment by contact frequency with adult children among older couples.

Neuropsychological Tests	More than Once a Month Contact			Less than Once a Month Contact		
	No. of Subjects with Lower Performance	OR	(95% CI)	No. of Subjects with Lower Performance	OR	(95% CI)
Cognitive impairment of husbands						
Total scores of SNSB-C	20	2.81	(0.94–8.40)	8	3.82	(1.06–13.75)
K-BNT	10	12.15	(1.5 × 10^−4^–9.9 × 10^5^)	4	1.58	(0.35–7.21)
RCFT	14	0.52	(0.08–3.24)	4	6.43	(1.01–41.10)
SVLT	22	0.32	(0.06–1.76)	6	3.64	(0.74–17.99)
DSST	15	8.2 × 10^2^	(6.2 × 10^−58^–1.1 × 10^63^)	4	2.18	(0.66–7.16)
COWAT: animal	20	2.44	(0.90–6.56)	5	6.04	(1.08–33.69)
COWAT: ‘ㄱ’	12	2.66	(0.89–7.96)	8	3.1 × 10^2^	(3.3 × 10^−51^–3.0 × 10^55^)
COWAT: animal+ ‘ㄱ’	15	2.3 × 10^2^	(1.1 × 10^−1^–4.6 × 10^5^)	4	4.87	(1.04–22.78)
KTMT	6	9.6 × 10^2^	(2.2 × 10^−13^–4.3 × 10^18^)	0	8.9 × 10^7^	(1.1 × 10^−34^–7.3 × 10^49^)
CWST	15	1.01	(0.35–2.95)	5	2.05	(0.62–6.83)
Cognitive impairment of wives						
Total scores of SNSB-C	10	0.48	(0.09–2.42)	4	0.80	(0.14–4.67)
K-BNT	10	2.6 × 10^−3^	(1.5 × 10^−44^–4.7 × 10^38^)	3	0.86	(0.28–2.68)
RCFT	18	1.64	(0.50–5.43)	3	1.29	(0.52–3.21)
SVLT	9	0.45	(0.13–1.58)	3	12.74	(3.5 × 10^−13^–4.7 × 10^14^)
DSST	5	4.4 × 10^−3^	(3.3 × 10^−75^–5.8 × 10^69^)	2	54.58	(8.7 × 10^−27^–3.4 × 10^29^)
COWAT: animal	12	0.56	(0.15–2.05)	5	0.70	(0.25–1.93)
COWAT: ‘ㄱ’	7	3.70	(4.3 × 10^−4^–3.2 × 10^4^)	4	2.7 × 10^−10^	(1.3 × 10^−24^–5.5 × 10^4^)
COWAT: animal+ ‘ㄱ’	12	1.31	(0.49–3.47)	4	0.69	(0.24–1.99)
KTMT	7	1.42	(0.48–4.22)	4	2.48	(7.8 × 10^−48^–7.9 × 10^47^)
CWST	11	0.91	(0.35–2.39)	5	6.1 × 10^2^	(1.6 × 10^−3^–2.4 × 10^8^)

K-BNT: Korean version of the Boston Naming Test, RCFT: Rey Complex Figure Test, SVLT: Seoul Verbal Learning Test, DSST: Digit Symbol Substitution Test, COWAT: Controlled Oral Word Association Test, K-TMT: Korean version of the Trail Making Test, CWST: Color Word Stroop Test.

**Table 6:** Beta coefficients for cognitive performance by contact frequency with adult children among older couples.

Neuropsychological Tests	More than Once a Month Contact			Less than Once a Month Contact		
	Beta	SE	*p*-Value	Beta	SE	*p*-Value
Cognitive impairment of husbands						
Total scores of SNSB-C	5.689	9.495	0.6564	−2.649	0.961	0.0074
K-BNT	−5.308	0.263	0.0315	−2.371	1.001	0.0206
RCFT	0.472	6.428	0.9533	0.486	0.994	0.6261
SVLT	1.017	16.044	0.9597	−1.289	0.999	0.201
DSST	0.543	12.978	0.9734	−1.154	1.037	0.2695
COWAT: animal	2.113	5.104	0.7501	−2.083	0.971	0.0354
COWAT: ‘ㄱ’	9.976	7.986	0.4298	−2.302	0.999	0.0242
COWAT: animal+ ‘ㄱ’	6.917	6.299	0.4703	−2.418	0.984	0.0165
KTMT	1.931	0.514	0.1657	−1.432	0.929	0.1275
CWST	−3.024	3.729	0.5662	−0.335	0.924	0.7177
Cognitive impairment of wives						
Total scores of SNSB-C	0.439	1.368	0.7504	0.538	1.020	0.6007
K-BNT	−0.154	1.271	0.9046	−0.314	0.775	0.6873
RCFT	0.679	1.462	0.6456	0.439	1.020	0.6696
SVLT	0.929	1.417	0.5175	0.808	0.858	0.3514
DSST	2.422	1.345	0.0821	−0.416	0.859	0.6306
COWAT: animal	−0.349	1.249	0.7818	0.515	0.902	0.5708
COWAT: ‘ㄱ’	−0.077	1.391	0.9564	−1.709	0.983	0.0892
COWAT: animal+ ‘ㄱ’	−0.203	1.378	0.8837	−0.650	0.970	0.5065
KTMT	1.221	1.422	0.3978	0.266	0.791	0.7386
CWST	−1.215	1.332	0.3694	−0.670	1.070	0.5346

K-BNT: Korean version of the Boston Naming Test, RCFT: Rey Complex Figure Test, SVLT: Seoul Verbal Learning Test, DSST: Digit Symbol Substitution Test, COWAT: Controlled Oral Word Association Test, K-TMT: Korean version of the Trail Making Test, CWST: Color Word Stroop Test.

## Data Availability

The data that support the findings of this study are available from the corresponding author, J.-y.M., upon reasonable request.
